# Extracts from a Lecture on the Laryngoscope

**Published:** 1864-10

**Authors:** George Johnson

**Affiliations:** Professor of Medicine in King's College; Physician to King's College Hospital.


*Extracts from a Lecture on the Laryngoscope.* Delivered at the Royal College of Physicians. By GEORGE JOHNSON, M. D., F. R. C. P., Professor of Medicine in King's College; Physician to King's College Hospital.

The laryingoscope is a small mirror, fixed on a stem, or handle, of convenient length. This mirror, having first been warmed to prevent the dimming of its surface by the patient's breath, is placed in such a position obliquely beneath the palate that, while it reflects the light from the mouth into the larynx, it reflects back an image of the larynx to the eye of the observer. There are various means, as we shall presently see, for throwing a strong light upon the mirror, but the laryngoscope is simply a small looking glass—a contrivance "whose end is to hold, as 'twere, the *mirror* up to nature."

Now it appears not a little remarkable that a method of exploring the larynx at once so simple and so effectual should not have come earlier into use—that it should have been reserved for the workers of the present to devise a plan by which literally a new light has been thrown upon a very common, painful, and fatal class of diseases.

Attempts to examine the larynx by means of a mirror have, at different times, been made independently by various experimenters. One of the first, if not the very earliest, of these attempts was made by a distinguished Fellow of this College—I mean Dr. Babington, who showed his instrument at a meeting of the Hunterian Society in March, 1829—i. e., thirty-five years ago. The instrument was essentially the same as that now in use, and the following description of it was published in the third volume of the *Medical Gazette*, p. 555. "It consisted of an oblong peace of looking-glass set in silver wire, with a long shank. The reflecting portion is placed against the palate, whilst the tongue is held down by a spatula, when the epiglottis and the upper part of the larynx become visible in the glass." The report adds that "the Doctor proposed to call it the *glottiscope*." Dr. Babington afterwards had his mirror made of polished steel, and in one he combined a tongue-depressor with the mirror. He also had one mirror of ovoid shape, which was convenient for use when the tonsils were enlarged. Dr. Babington tells me that he was in the habit of illuminating the throat by reflecting the light of the sun from a mirror held in the left hand. It was long after Dr. Babington had published the account of his glottiscope that Mr. Liston, in his "Practical Surgery," (1840,) referred to the use of a dentist's mirror for obtaining a view of the glottis.

MM. Trousseau and Belloc, in a treatise on Laryngeal Phthisis, which was published in the year 1837, refer to a *speculum laryngis*. It was made by M. Selligue, an ingenious mechanic, who had himself suffered from laryngeal phthisis. The instrument consisted of two tubes, through one of which the light was thrown on the glottis, while through the other the image of the glottis was reflected from a mirror placed at its gutteral extremity. The authors state that the instrument was very difficult of application, and that no one person in ten could bear its introduction.

The late Mr. Avery worked long and successfully in the construction of a laryngoscope and other instruments for the examination of internal organs, but he published nothing on the subject.*

In the year 1844 the late Dr. Warden invented a prysmatic speculum, with which he succeeded in seeing disease of the glottis in two cases.†

It is a well-known fact that the first experimenter who succeeded in obtaining a view of his own larynx is a distinguished professor of music in this town, M. Garcia. M. Garcia had long studied the anatomy and physiology of the larynx as the organ of voice, and he had a great desire to see the movement of the living larynx. At length he obtained the desired object by a very simple plan. Standing with his back to the sun, he held a looking-glass in his left hand before his face; the sun's rays were reflected by the glass into his open mouth. Then he introduced a dentist's mirror, previously warmed, into the back of his mouth, and thus he saw the reflection of his larynx in the looking-glass.

M. Garcia gave the results of his observations in a very interesting paper entitled "Physiological Observations on the Human Voice," which was published in the "Proceedings" of the Royal Society in the year 1855. This paper was destined to be the germ of further important observations and discoveries. It became known to Dr. Turck, of Vienna, and it induced him to use the laryngeal mirror in the wards of the General Hospital there during the year 1857. Towards the end of that year Dr. Turck lent his mirror to Dr. Czermak, who set to work with great zeal and energy. He soon made the important step of adopting the large ophthalmoscope reflector as a means of concentrating artificial light, thus making the laryngoscope available at all times as a means of inspecting the larynx, and of guiding the hand in the application of local remedies. Czermak soon saw, as he says, the practical value of the instrument, and he has been most energetic and most successful in his efforts to secure its recognition by the whole civilized world.

It appears to me that, without injustice to those who had pre-

---

* Introduction to the Art of Laryngoscopy. By Dr. Yearsley, 1862.

ceded him, Garcia's claims to originality in the matter of auto-largyngoscopy being obviously quite distinct and indisputable—Czermak may be considered to be the discoverer of the art of laryngoscopy *in its application to the diagnosis and treatment of disease.* He was also the first to practice the kindred art of *rhinoscopy.*

Sydney Smith, in discussing the rival claims of discoverers, has said, "That man is not the first discoverer of any art who first says the thing; but he who says it so long, and so loud, and so clearly, that he compels mankind to hear him—the man who is so deeply impressed with the importance of his discovery that he will take no denial; but at the risk of fortune and fame, pushes through all opposition, and is determined that what he thinks he has discovered shall not perish for want of a fair trial." On grounds such as these—not of priority in time, but in persevering and successful efforts to render the method practically available—Czermak has established strong claims to be considered the *discoverer*, as he has unquestionably been the great *improver* and the great *teacher*, of the arts of laryngoscopy and rhinoscopy, in their application to the diagnosis and treatment of disease.*

I propose now to describe the *method of using the laryngoscope.*

And, first as to the mode of illuminating the throat. The plan which is generally adopted is to reflect the light of the sun or of a lamp into the throat by means of a concave mirror, which is fixed on the forehead or in front of one eye of the operator.

The operator always sits opposite to the patient. When sunlight is used, the patient is placed with his back to the sun. When a lamp is employed, it is placed usually to the right side of the patient's head and on the same level, or a little above. In using artificial light, it is unnecessary to darken the room more than may be done by simply drawing down a blind, so as to lessen the glare of daylight. Now the question arises, should the reflector be perforated and placed in front of one eye, so that we look through it into the patient's throat, or is it better placed on the forehead just above the eyes? in which case it is unnecessary to have the mirror perforated. *I believe that the best position for the reflector is above both eyes and not in front of one,* and as this is a point of considerable importance, I must give the reasons for my belief.

With the reflector on the forehead we avoid the discomfort and inconvenience resulting from the effort required to keep one eye applied to the opening in the mirror. We have the free and unimpeded use of both eyes, and we consequently find it much easier to direct the light into the patient's throat, to introduce the laryngeal mirror, and to practise any other manipulation that may be required either for diagnosis or treatment. Another incidental advantage attending the position of the reflector on the forehead is, that we thus get a more extended movement of the reflector in all directions. This free movement enables us readily to change the direction of the light when we are examining our patient, and it also facilitates a very simple mode of auto-laryngoscopy of which I shall presently have to speak. The question then arises are there advantages to be gained by looking through a perforated reflector which in any degree compensates for its manifest inconveniences? I know of none, and I believe that none exist. The practice of using a perforated reflector was borrowed from the ophthalmoscope; but the conditions which attend the exploration of the interior of the eye through the small opening of the pupil are very different from those which exist when we are looking through the wide open mouth at an image of the larynx reflected from a mirror of considerable size. In the latter case there is nothing gained by looking through the centre of a perforated reflector. I have fully tested this, not only in the examination of the larynx, but also by an experiment of this kind. Place a stethoscope, with the ear-piece downwards, on the table in front of you. Hold a laryngeal mirror obliquely over the upper end of the stethoscope, so as to reflect the interior of the tube, throwing the light of a candle on the mirror by means of the concave reflector placed at one time on the forehead, at another in front of one eye. You will find that, as regards the facility of illuminating the interior of the tube and seeing its image in the mirror, the position of the reflector makes not the slightest difference.

I have met with very few persons who have tried both methods, fail to appreciate the great convenience and advantage of having the reflector on the forehead rather than in front of one eye. Some who have become accustomed to the latter plan are unwilling to change it. Czermak not only keeps the reflector in front of the right eye, but he holds the apparatus between his teeth—a practice in which he has found very few imitators. M. Garcia* states, with regard to the use of a perforated mirror, that he tried it in order that Drs. Sharpey and Williamson might observe his larynx while he experimented upon himself. He found, however, that this was not attended by any marked advantage. They could see the reflected image of his larynx as well as by looking over the top of the mirror as by looking through its perforated centre.

I made the same observation when looking into Czermak's throat while he was using his auto-laryngoscopic apparatus; I could see his larynx as well by the side of the reflector as through its centre. When I am examining the larynx of a patient, if I wish to make the parts visible to another, I can readily do this by turning the face of the laryngeal mirror slightly towards one side, and directing the observer to look over my shoulder at the mirror in the throat. In order to see the image in the larynx it is unnecessary that the eye should be even *near the margin of the reflector*, much less is it necessary that the eye should look through the *centre of the reflector.*

The reflector when in front of the eye, therefore, being a source of much discomfort and inconvenience, without any compensating advantage, is better placed on the forehead just above the eyes.

The *faucial or laryngeal mirror* is made of different forms—square, with the angles rounded off, circular or oval. The form of the mirror is of little consequence. I find, however, that a circular mirror irritates the back of the pharynx less than a square one; I therefore prefer the circular form. Silvered glass mirrors are to be preferred to those made of steel or other metal. Metallic mirrors soon lose their polish, and they quickly cool, and thus become dimmed by the breath.

The mirror is to be warmed by holding it over the lamp or by dipping it into warm water. Its temperature should be tested by bringing it in contact with the cheek or the hand of the operator. It should be warm enough to prevent its being dimmed by the patient's breath.† There are two reasons for not over-heating the mirror—first, the patient's mouth will be burned; and, second, the silvering of the mirror will be spoiled.

The mirror is to be held like a pen, between the thumb and two fingers, and introduced so as to slightly raise the uvula and soft palate. Care must be taken to avoid touching the tongue, and as

---

* Dr. Christie, of Aberdeen, claims for Levret the original suggestion of such an instrument. L'art des Accouchements, Paris, 1743.

*Notices sur l'Invention du Laryngescope, par Paulin Richard. Paris, 1861. p. 14.

†Dr. Buzzard (Lancet, August 1864,) advises the application of a little dilute glycerine to prevent the aqueous deposit on the mirror.

much as possible the back of the pharynx, with the mirror, these being the most sensitive parts within the mouth. The hand of the operator may be kept steady by resting the third and fourth fingers on the chin of the patient.

I have said that we must not touch the tongue with the mirror; but how is this to be avoided? You will find that, very generally, as soon as the mirror is introduced between the teeth, the tongue involuntarily rises towards the roof of the mouth, so as to come in contact with the mirror and obstruct the view; and, in fact, the tongue is one of the most frequent and most serious impediments in the way of laryngoscopy. There are various modes of dealing with this unruly member.

In some few cases the patient has sufficient control over the tongue to hold it down by a voluntary effort while the laryngeal mirror is being introduced. This power, however, is rarely acquired until after a considerable amount of practice, and in most instances the tongue has to be kept out of the way by some mechanical means. The plan which usually succeeds best is to hold the tip of the tongue between the thumb and the forefinger, and to draw it gently forward over the lower teeth. This may be done by the operator with his left hand, or by the patient, the thumb and finger which hold the tongue being covered by a cotton glove, or by a towel or handkerchief.

In some cases a *metallic tongue-depressor* may be used with advantage, or the tongue may be pressed down by the fore-finger of the operator's left hand. But it will usually be found that one effect of depressing the tongue in front is to push it backwards at the base, so that it nearly or quite touches the back of the pharynx, thus intercepting the light; while another effect is to make the tongue arch upwards, so as nearly to touch the roof of the mouth. This arched position of the tongue obstructs the passage of the light to and from the larynx; often, too, it brings the tongue in contact with the mirror, and this excites nausea. For these reasons the attempt to depress the tongue is usually less successful than its gentle traction forwards.

I have before said that the laryngeal mirror is to be introduced so as slightly to raise the uvula and soft palate. The uvula must not be allowed to project below the mirror. The end of a long uvula hanging below the mirror has its image reflected in the glass, and this obscures the view of the larynx. The uvula and the soft palate are the least sensitive parts with which the mirror can come in contact. The posterior wall of the pharynx is usually more sensitive, and care should be taken to disturb it as little as possible. Frequently, however, the pharynx bears the touch of the mirror as well as the uvula and soft palate.

The mirror being placed in an oblique position below the palate, we usually at once obtain a view of the larynx. A little practice will enable you to make such changes in the position of the mirror, or of the patient or in the direction of the light, as may be required to bring the parts fully into view. It should be borne in mind that the larynx, as it appears in the mirror, is reversed; so that we get the same view as we have when, examining the larynx after death, we look at it from behind. The arytenoid cartilages are nearest to the eye; the insertion of the vocal cords into the thyroid cartilage is more distant. We also see the anterior wall of the trachea as if we were looking into the tube from behind. We see that during inspiration the glottis is a wide triangular opening of considerable size, the vocal cords being of a pearly white color. During speaking—as in pronouncing the syllable "eh"—the glottis closes, and the cords vibrate with the impulse of the expired air.

It is important to practice the introduction of the laryngeal mirror with the *left* hand as well as with the right. In applying local remedies to the larynx the patient is instructed to manipulate his own tongue while the operator, holding the mirror with the left hand so as to obtain a view of the larynx, uses his right hand for the introduction of the brush or other instrument.

But how does the throat bear the contact of the mirror? Does not its introduction excite retching and cough and dyspnœa, and other unpleasant sensations? These questions are often asked by those who have had no experience in laryngoscopy; but those who have experience are unanimous in declaring that, in the great majority of cases, none of these unpleasant results attend the introduction of the mirror into the fauces. In some instances, however, we meet with difficulties in the use of the instrument. I will briefly refer to some of these, and will give some hints as to the best mode of meeting them.

First, then, some persons have a propensity to throw the tongue forcibly upwards towards the roof of the mouth; and they do this with a provoking pertinacity just as the mirror is being introduced between the teeth. This position of the tongue offers a serious impediment to the introduction of the mirror, and the obstruction is greater in proportion to the size of the rebellious tongue. It is usually a result of nervousness on the part of the patient, and is sure to be made worse by any appearance of petulence in the operator. The better plan is to endeavor to reassure the patient. Sometimes the occupation of holding his own tongue has a good effect by diverting his attention, and occasionally, while he is holding the tip of his tongue, you may depress the dorsum with a spatula or with your finger. In some instances, after making one or two attempts, it is better to defer examination to a future day. After two or three sittings, there is usually less nervousness, and the tongue comes more under control.

[Dr. Watson, after hearing this lecture, told me that in the case of patients who have this tendency to arch up the tongue, and so to prevent the examination of the fauces, he directs them to practise by sitting in front of a looking-glass with the mouth open. The inspection of the tongue, while they are endeavouring to acquire the power of controlling its movements, is found to be a great assistance.]

Another impediment to the examination of the larynx results from unusual sensitiveness of the fauces, so that the touch of the mirror excites contraction of the pharynx and retching. This excessive sensibility is common when the fauces are in a state of inflammatory congestion; so that, seeing the throat engorged and red, we may anticipate a difficulty in the examination of the larynx. There are two modes of lessening the sensibility of the throat in such cases. One is, to direct the patient to keep a lump of ice in his mouth for ten or fifteen minutes before the examination, and as the ice melts, to swallow the cold water. Another, and I think a more effectual plan, is to put twenty drops of chloroform on a handkerchief and let him inhale it for a minute: I have found this successful in quieting the most irritable throats, and that without rendering the patient in the least degree drowsy or uncomfortable. The bromide of potassium, when swallowed or used as a gargle, has long been supposed to have the effect of lessening the reflex sensibility of the fauces, but in the few cases in which I have tried it for this purpose it has appeared to be quite inert; Semeleder states, too, that he has not obtained the desired result from this salt.

It will usually be found that the repeated introduction of the faucial mirror at intervals of a day or two has the effect of lessening the sensibility of the throat, so that after a short time the most sensitive throat becomes tolerant of the mirror.

I have found that patients laboring under acute laryngitis and other organic diseases which are attended with much suffering usually bear the examination well, and often better than others who have but trifling ailments, or none at all. The man who is threatened with suffocation will submit to any proceeding which affords him hope of relief, and the distress in his larynx is so great that he is scarcely conscious of the trifling irritation caused by the faucial mirror; so true is it that

> "Where the greater malady is fixed
> The lesser is scarce felt."

Enlargement of the tonsils may render the examination of the larynx difficult or impossible. A small mirror may be used when the enlargement is not excessive; but if the tonsils are so much enlarged as to touch each other, a laryngoscopic examination is impracticable.

The epiglottis is some times very long, and projects obliquely downwards and backwards, so as to make it impossible to throw the light beneath it, and to get a view of the larynx. The arch of the epiglottis too, is sometimes so contracted as to obstruct the entrance of the light.

Semeleder* gives us the result of his experience that in about 25 per cent. of adults he got a perfect view of the larynx easily at the first examination; in about 5 per cent. it was impossible to see the larynx at all; in the remainder he succeeded more or less completely after repeated examinations. In children from two years of age and upwards the proportion of failures is much greater.

In the practice of auto-laryngoscopy, whether in the examination of one's own larynx or that of others, it is of primary importance that the operator should have the power of readily changing the direction of the light, so as at once to adapt it to the varying position of the body, which is often required for the thorough exploration of the larynx. A feebler light which can readily be reflected in any required direction, is of more practical value in laryngyscopy than a stronger light which is fixed.

Some laryngoscopists on the continent, and Dr. Walker of Peterborough, do not use the reflector for the purpose of lighting the throat, but in place of it they get a direct illumination of the fauces by means of a concentrated fixed light. A globular bottle of water in front of a lamp is used as a powerful condensing lens. In this way, certainly, a very bright light is obtained; but the objections to this mode of illumination are—1st, that the apparatus is clumsy, and cannot be carried about; and, 2nd, the chief objection is that the direction of the light cannot be readily and instantaneously made to follow the movements of the patient's head The fact of the light moving with the movements of the operator, which some consider an objection to the method of illuminating the throat by means of the reflector on the forehead, does, in fact, constitute one of its chief advantages.

With regard to the source of the light, I find it not difficult to see and to demonstrate my own layrnx, as well as to examine the larynx of another, by the light of an ordinary candle; but a bright light renders the examination much easier and more satisfactory. The best artificial light is a moderator lamp, or an argand gas-burner. The light may be much intensified by placing a metallic reflector behind the lamp, and a bull's-eye condensor at the proper focal distance in front, the flat side of the lens being next the lamp. I find that with a single bull's-eye condensor I get a better light than with Tobold's condensor, which consists of three lenses in a brass tube, and which is a more cumbersome as well as a more costly apparatus.

All observers agree in opinion that the light of the sun, when it can be obtained, is the best means of illuminating the throat. The patient sits with his back to the sun, and the operator directs the light into the throat by means of a reflector. For this purpose the reflector need not be concave; a flat surface will give sufficient light. In using a concave reflector with sunlight, you must be careful not to burn the throat by concentrating the rays into a focus. *Solar* caustic, be it remembered, may be made more powerful than *lunar* costic. [Since this lecture was given I have found that the best mode of using sunlight in laryngoscopy is to place a looking glass in such a position that it shall deflect the sun's rays on the frontal reflector, but leave the eyes of the operator in the shade. In this way we avoid the serious inconvenience which results from exposing the eyes to the direct rays of the sun. Both the patient and the operator are in the shade, a column of light being turned upon the frontal reflector by the looking-glass.] With sunlight it is not absolutely *necessary* to use the frontal reflector. The patient may face the sun, so that the rays fall directly upon the laryngeal mirror. But here, again, the advantage of the reflector consists in the facility with which it enables you in a moment to change the direction of the light

The reflector on the forehead is a very useful means of lighting the throat for the purpose of examining the tonsils, palate, and pharynx. Placing a lamp or a candle by the side of the patient, or using sunlight when it is available, the operator, with the reflector on his forehead, throws the light into the throat, and has both his hands free to depress the tongue and to apply caustic or other local remedies. In cases of diphtheria and scarlatina, by this method of illumination a thorough examination of the throat can be made in a much shorter time than by the ordinary method, and without the necessity of raising the patient's head from the pillow. The operator in this way runs less risk of infection from inhaling the patient's breath, or from the morbid secretions being coughed into his face.

In the presence of a learned assembly such as I have now the honor to address, it is scarcely necessary to assert, that if in the laryngoscope we have an important aid in the diagnosis of laryngeal disease, such aid is by no means superfluous or uncalled for. The experience of every practitioner will enable him to recall cases in which there has existed the most painful doubt as to the nature of disease within the larynx. Mr. Porter, writing in the year 1837, says, "How is a man of experience. when he meets with a case of laryngeal disease, to know whether it is caused by an œdematous condition of the sub-mucous tissue—by a chronic thickening of the mucous membrane itself—by laryngeal ulceration—by destruction of the cartilages—by the presence of abscess or tumor, or by another of those numerous affections which dissection so frequently shows us to be the occasion of death? And he suggests that "perhaps by reason of the difficulty of the subject, it will be long before we possess the same accuracy of information with reference to affections of the windpipe that has been attained in other diseases." What, now, has been the effect of that simple contrivance, the laryngeal mirror? May it not be said without exaggeration that it has rendered the diagnosis of the diseases of the larynx more simple and more certain than the diagnosis of the diseases of any other internal organ? In fact the larynx has ceased to be an internal organ, in the sense of being hidden from view, for it has been brought within the range of vision. And the answer to Mr. Porter's question is simply this, that the man

* Die Laryngoskopie und ihre verwerthung fur die Artzliche Praxis. Von Dr. Friedrich Semeleder. Wien, 1863.

14

of experience has now only to *look* into the larynx, and he will *see* what is the form of a disease with which he has to deal.

In my next lecture I propose to give some illustrations of the valuable aid which the laryngoscope is capable of affording in both the diagnosis and the treatment of disease. During the few minutes that remain to-day, I propose to advert very briefly to the subject of rhinoscopy.

*Rhinoscopy.*

Czermak, in his first publication on the laryngoscope, pointed out that the same method of examination was applicable to the inspection of the posterior surface of the soft palate, the posterior openings of the nasal fossæ, and the superior parts of the pharynx.

In the practice of rhinoscopy the patient should sit erect, without throwing the head back, while the light is thrown into the mouth by the frontal reflector. The tongue is to be kept down by means of a metallic depressor, which may be held either by the operator or by the patient. A small mirror is required, and it is better made of glass than of metal, on account of the rapidity with which a metallic mirror cools and condenses vapor on its surface. I have two circular mirrors, which I find very convenient for rhinoscopy, one the size of a three-penny piece, the other the size of a sixpence.

When you are about to introduce the mirror, the patient should be directed to breathe quietly. A deep inspiration draws the uvula and soft palate upwards and backwards, and so interferes with the examination. The mirror is to be introduced by the side of the uvula, beneath the palate, with its surface directed upwards and forwards. The facility with which the examination can be made depends mainly upon the space which exists between the palate and the posterior wall of the pharynx. When the interval is a moderately wide one, the mirror can be introduced without touching the uvula or palate, and the posterior openings of the nasal fossæ, the turbinated bones, the opening of the Eustachian tube, the septum narium, the roof of the pharynx,—all these parts may be distinctly seen.

In some cases the examination is facilitated by drawing the uvula and palate forwards by means of a blunt hook; but this is better avoided if possible, for it is always attended with much discomfort, and frequently the contact of the hook excites contraction of the palate, which is then drawn upwards and backwards so as completely to obstruct the view. The most successful rhinoscopic examinations that I have made, have been accomplished without touching the uvula and soft palate. Very valuable information may sometimes be obtained by rhinoscopy.

Last year I was consulted by a gentleman, twenty-four years of age, who had complete obstruction of the right nostril. It had commenced two years ago, after a severe cold; and it had steadily increased until, at the end of about a year, it was so complete that he was unable by an effort either to inspire or to expel air through the right nostril. The left nostril remained pervious, but in consequence of the obstruction on the right side the patient habitually kept his mouth open, respiration being impeded when the mouth was shut; and the voice had somewhat of a nasal character. Examination of the nostril in front discovered no obstruction, nor was any abnormal appearance visible on examination of the palate and pharynx in the ordinary way through the open mouth.

He had a throat favorable for rhinoscopy: a small uvula, with the soft palate at some distance from the back of the pharynx, so that the mirror could be introduced without disturbing these parts. The left nasal fossa was quite normal, but the right was seen to be completely obstructed by a tumour, apparently of globular form, having a slightly granular surface and a yellowish-green color. It touched the floor and septum of the nose; and externally it pressed upon and concealed the turbinated bones. I could touch the tumour with a bent probe introduced behind the palate. I now asked my friend and colleague, Mr. John Wood, to see the patient with me, and to devise a plan for removing the tumour. He introduced a pair of slender curved polypus forceps through the anterior opening of the nostril, grasped the tumour, and as he was drawing it forward there was a sudden rush of a glairy fluid, like white of egg, and some membranous shreds came away between the blades of the forceps. The patient felt immediately that the obstruction was gone. On rhinoscopic examination the tumour had disappeared; the turbinated bones were plainly visible; and on the under side of the middle turbinated bone there was an abraded surface, from which, apparently, the tumour had been torn. The tumour had evidently been a globular cyst containing fluid. On the second day after the operation a portion of the cyst wall came away. This I have preserved. It is smooth on its inner concave surface, but uneven on its outer surface, by which apparently it had been attached to the mucous membrane. During the first few days after the operation, the abraded surface of the mucous membrane was covered by a purulent secretion; this quickly healed. The patient has lost all sense of obstruction in the nostril; he can breathe comfortably with the mouth closed; and the voice has recovered its natural tone.

The practical value of rhinoscopy in this case can scarcely be called in question. It is doubtful whether by any other mode of examination the position and nature of the tumor could have been determined with sufficient certainty to warrant an operation for its removal. I was relating this case to a friend, who remarked that my patient had more reason to congratulate himself than one about whom he was consulted. One nostril was obstructed, and it was supposed that a polypus was the cause of obstruction. A surgeon had made an unsuccessful attempt to remove the supposed polypus by the forceps; this caused much suffering; and it was at last discovered that the obstruction was due to thickening of the turbinated bones.

Czermak, in the last German edition of his work,* gives a good illustration of the value of rhinoscopy in correcting an erroneous diagnosis. A young man, deaf on the left side, was found to have a tumour at the back of the nostril, which conveyed to the finger the impression of a polypus. An operation was contemplated, but a rhinoscopic examination discovered a tapering swelling of the mucous membrane, nearly as thick as the finger, surrounding the orifice of the left Eustachian tube; also great swelling of the middle and inferior turbinated bones; but no polypus, nor any tumour which an operation could have removed or lessened.

